# Functional and Proteomic Investigations Reveal Major Royal Jelly Protein 1 Associated with Anti-hypertension Activity in Mouse Vascular Smooth Muscle Cells

**DOI:** 10.1038/srep30230

**Published:** 2016-07-22

**Authors:** Pei Fan, Bin Han, Mao Feng, Yu Fang, Lan Zhang, Han Hu, Yue Hao, Yuping Qi, Xiaozhen Zhang, Jianke Li

**Affiliations:** 1 Institute of Apicultural Research/Key Laboratory of Pollinating Insect Biology, Ministry of Agriculture, Chinese Academy of Agricultural Sciences, Beijing 100093, China; 2 College of Biological Engineering, Henan University of Technology, Zhengzhou 450001, China

## Abstract

Vascular smooth muscle cells (VSMCs) are a major cell type of the arterial wall and their functionality is associated with blood pressure regulation. Although royal jelly (RJ) has reported effects on anti-hypertension, the mechanism of blood pressure regulation by major royal jelly protein 1 (MRJP1), the most abundant RJ protein, is still unknown. The *mrjp1* gene was inserted into mouse VSMCs to investigate how MRJP1 influences VSMC functionality by functional and proteomic analysis. The expression of MRJP1 in VSMCs significantly reduced cell contraction, migration, and proliferation, suggesting a potential role in decreasing hypertension via action on VSMCs. These anti-hypertension activities were further observed in the changes of the proteome setting of mouse VSMCs. Among 675 different proteins after MRJP1 expression, 646 were down-regulated and significantly enriched in pathways implicated in VSMC contraction and migration, which suggest MRJP1 lowers VSMC contraction and migration by inhibiting muscle filament movement. The down-regulated proteins also enriched pathways in proliferation, indicating that MRJP1 hinders VSMC proliferation by reducing the supply of energy and genetic material. This is the first report integrating MRJP1 into VSMC, revealing the function and mechanism correlated with anti-hypertensive activity. This offers a therapeutic potential to control hypertension by gene-therapy using bee-products.

Cardiovascular diseases (CVDs) severely jeopardize human health and affect the largest number of people (17.5 million deaths per year) among all diseases worldwide^1^. Hypertension in particular is one of the major causes for CVDs, and is the leading risk factor for global disease burden^2^. Vascular smooth muscle cells (VSMCs), the main cellular constituents of blood vessels, are localized in the tunica media and control vascular contraction/relaxation, thus determining blood pressure^3^. Normally, there are two phenotypic VSMCs in blood vessels, proliferative or contractile. The differentiation of VSMCs from proliferative to contractile type usually occurs by the high expression of specific marker genes, including smooth muscle actin α (αSMA), smooth muscle protein 22α (SM22) and calponin^4^,^5^. Therefore the VSMC contractibility can be determined by the expression levels of these protein markers. Additionally, the VSMC proliferative ability is closely related to blood pressure regulation. The increasing number of VSMCs in blood vessels is regarded as a major contributor to the thickness of the blood vessel wall in vascular remodeling, which is a major driving force of increased contraction in blood vessels, thus in turn elevating the blood pressure^6^,^7^. Consequently, VSMC contraction and proliferation are referred to as the key elements that regulate blood pressure. Moreover, abnormal proliferation and migration of VSMCs can eventually result in atherosclerosis^8^,^9^, another severe cardiovascular disease that also has a close relationship to hypertension. It is known that blood pressure can also be regulated by endogenous and/or exogenous molecules *in vivo*^10^,^11^. This provides potential venue to regulate VSMC contraction, proliferation, and migration using specific molecules, such as genes and proteins, for the safe and effective treatment of hypertension.

Royal jelly (RJ), a well-known functional food secreted by the hypopharyngeal and mandibular glands of honey bee workers, has a documented, wide range of biological applications for promoting human health, including anti-hypertension, anti-tumor, anti-infectious and anti-oxidative effects^12^. It is known that proteins are the main constituents of RJ, accounting for more than 50% of its dry weight. Particularly, major royal jelly proteins (MRJPs) 1–9 compose over 80% of the proteins, among which MRJP1 is of the highest abundance, consisting of approximately 48% of the water soluble proteins in RJ with a molecular weight of 55–57 kDa^13^,^14^. MRJP1 is important for honeybee biology and human health promotion. For honeybees, the continuous ingestion of RJ is required for the formation of a queen from the larval stage, in which MRJP1 plays the dominant role in determining whether the larvae develop into a queen or worker bees^15^. In regard to the regulation of hypertension, it is reported that enzymolysis fragments of RJ proteins are capable of reducing blood pressure in hypertensive animal models by oral administration^16^,^17^, however, the specific components are still unknown. Only very recently has MRJP1 been reported as a potential candidate for regulating blood pressure by *in vitro* assay^12^, but how exactly MRJP1 regulates blood pressure at the cellular and molecular levels still remains unknown.

Lentiviral vector, a commonly-used, robust tool in delivering specific genes into mammalian cells, is characterized by high efficiency, low toxicity and stable targeting^18^. Thus, the vector is usually applied in gene therapy for the treatment of human diseases^19^. To express MRJP1 in VSMCs using lentiviral vector may be an alternative approach to reduce the protein break down in gastrolintestinal tract and cell metabolism by oral and *in vitro* administration of MRJP1. Hence, the present work was performed on the basis of integrating the *mrjp1* gene (*A. m. ligustica*) into VSMCs to express MRJP1 to gain insight into the function and mechanism of how MRJP1 acts on blood pressure regulation. This study may offer a potentially new avenue to treat the hypertension disease with gene therapy derived from natural bee-products.

## Results

### Lentivirus can deliver *mrjp1* into mouse VSMC genome

**Figure 1:**
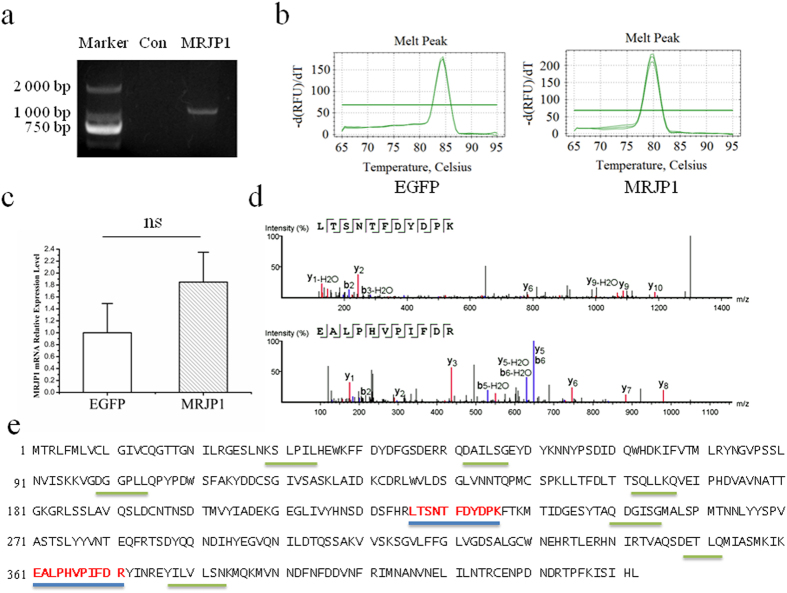
Delivering *mrjp1* gene into mouse VSMC genome by lentiviral vector and its correctly expression in VSMC. **(a)** PCR amplification of CDS of *mrjp1* gene was visualized on agarose gel by electrophoresis from the transduced VSMCs. **(b)** Melting curves show that both EGFP and *mrjp1* mRNAs are specifically amplified by real time RT-PCR from *mrjp1* integrated VSMCs. **(c)** Relative expression level of mRNA of *mrjp1* is higher than that of EGFP but without statistical significance from three replicates. **(d)** Two unique peptides (LTSNTFDYDPK and EALPHVPIFDR) of MRJP1 are identifed in *mrjp1* integrated VSMCs by LC-MS analysis. **(e)** The peptides identified in *mrjp1* integrated VSMCs are matched to the full sequence of MRJP1 (*A. m. ligustica*). Peptides with blue bottom line are unique in MRJP1 while the ones with grey bottom line are non-unique.

In an effort to deliver *mrjp1*into the mouse VSMC genome, the coding sequence (CDS) of *mrjp1 (A. m. ligustica*) was inserted into the multiple cloning sites of the lentiviral vector as shown in Fig. S1a. The successful expression of EGFP is an indicator of transducing VSMCs by lentivirus. In both control and MRJP1 expressing VSMCs, EGFP was observed by fluorescence microscopy 48 h after transduction (Fig. S1b). To confirm *mrjp1* was integrated into the VSMC genome, the CDS of *mrjp1* was amplified by PCR and visualized on agarose gel with correct size. As expected, no band was found in the control VSMCs (Fig. 1a). To certify that *mrjp1* can be transcribed in mouse VSMCs, real time RT-PCR analysis was applied. Notably, a melting curve was performed to ensure the specificity of *mrjp1* mRNA amplification (Fig. 1b), indicating that the CDS of *mrjp1* was transcribed into mRNA. Compared to EGFP, the mRNA expression level of MRJP1 was higher but without a statistically significant difference (Fig. 1c). The low MRJP1 signal recovered from EGFP cells displayed a different melting temperature, indicating that it was not derived from MRJP1 sequences. In the VSMCs that CDS *of mrjp1* were delivered, two unique peptides (LTSNTFDYDPK and EALPHVPIFDR) (spectra of these two peptides shown in Fig. 1d) were matched to the protein sequence of MRJP1 by LC-MS/MS analysis (Fig. 1e). The identification of MRJP1 reached the confidence level more than 99% at both peptide and protein levels by false discovery rate (FDR) calculation using the PEAKS search program, showing that the gene of *mrjp1* can be expressed in VSMCs. In contrast, no such peptides of MRJP1 were found in the control VSMCs, demonstrating the fact that mRNA of the *mrjp1* gene can be translated into the corresponding protein of MRJP1 in VSMCs. In sum, the CDS of the *mrjp1* gene was able to be integrated into the VSMC genome and transcribed into mRNA that was finally translated to MRJP1.

### Expressing MRJP1 inhibits mouse VSMC contraction

**Figure 2:**
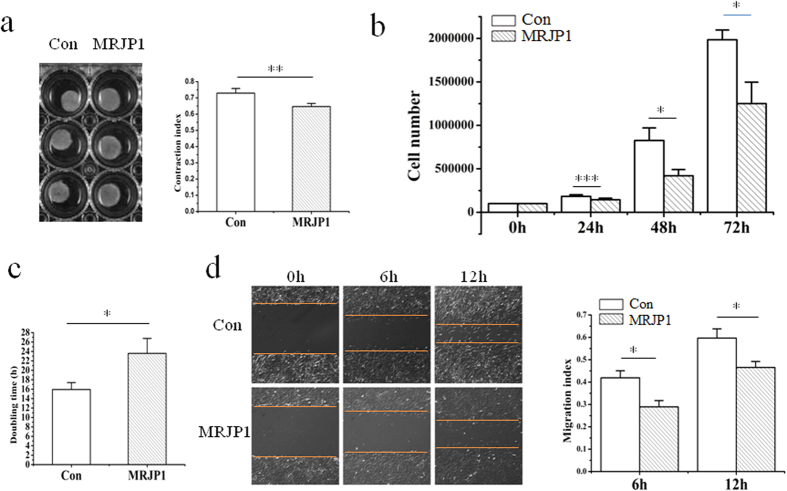
MRJP1 expressing VSMCs show reduced contractive, migrating and proliferative phenotypes. **(a)** Contraction index is significantly reduced in MRJP1 expressing VMSCs compared to control from three independent experiments by collagen gel assay (n = 3, mean ± S.D). Results are shown as mean ± S.D and **p < 0.01. **(b)** Cell are counted at 24 h, 48 h and 72 h, respectively, to compare the proliferative rates between control and MRJP1 expressing VSMCs (n = 3, mean ± S.D). *p < 0.05, ***p < 0.001. **(c)** Doubling time (*td*) of MRJP1 expressing VSMCs is significantly delayed compared to that of controls. *td* of VSMCs was determined using *ln(N*_*48h*_*/N*_*0h*_) = *k(t*_*48h*_ − *t*_*0h*_) and calculating *td* = *ln 2/k* when cell number doubling for a given k (n = 3, mean ± S.D). *p < 0.05. **(d)** Migration index is significantly decreased by the expression of MRJP1 in VSMCs at 6 h and 12 h, respectively by wound healing assay (n = 3, mean ± S.D). *p < 0.05.

**Figure 3:**
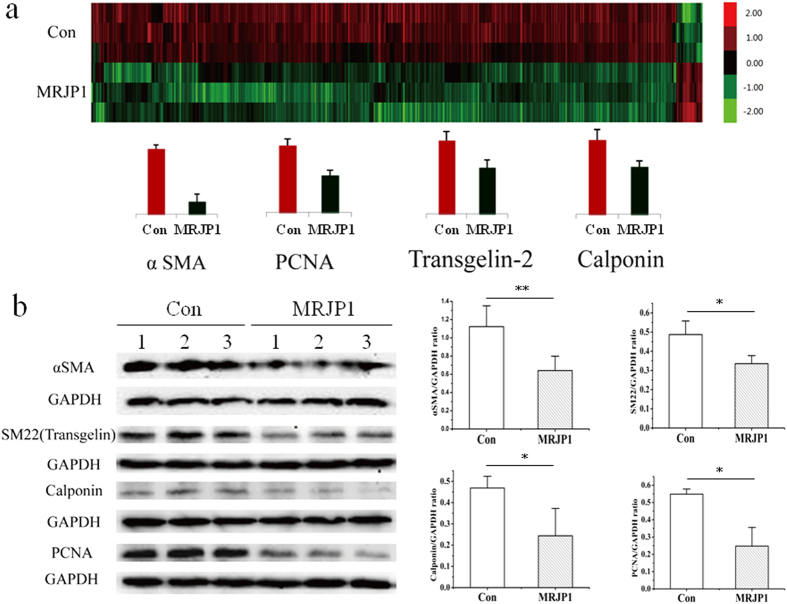
MRJP1 expression changed mouse VSMC protoeme setting. **(a)** Clustered heatmap displayed differentially expressed proteins between control and MRJP1 expressing mouse VSMCs with three replications in each group. **(b)** The expressions of αSMA, SM22, calponin and PCNA in MRJP1 expressing VSMCs are significantly reduced by Western blotting analysis compared with controls, the band intensities were normalized to GAPDH (n = 3, mean ± S.D) from three independent experiments. *p < 0.05, **p < 0.01.

To determine the roles of MRJP1 regulating VSMC contractibility, a collagen gel contraction assay was performed on control and MRJP1 expressing VSMCs. The contraction index of MRJP1 expressing VSMC was significantly lower than that of the control, or the contractibility of mouse VSMC was reduced by MRJP1 (Fig. 2a). Moreover, the expression levels of αSMA, SM22 and calponin (Fig. 3b) by Western blotting analysis showed that all the VSMC markers related to determining VSMC contraction and differentiation were found significantly down-regulated in MRJP1 expressing VSMCs. In addition, the expression of αSMA was significantly down-regulated by the expression of MRJP1 in VSMCs through an immunofluorescence assay (Fig. S3). In short, the contractibility was significantly inhibited by the expression of MRJP1 in VSMCs.

### Expressing MRJP1 decreases migration of mouse VSMCs

To compare the migrating abilities of VSMCs between control and MRJP1 expressing VSMCs, a wound healing assay was applied. The migration indices of MRJP1 expressing VSMC were significantly reduced after 6 h and 12 h relative to those in the control, respectively (Fig. 2d), indicating MRJP1 could suppress the migrating ability of mouse VSMC.

### Expressing MRJP1 hinders proliferation of mouse VSMCs

To examine the function of MRJP1 in regulating VSMC proliferative ability, a cell number counting assay for control and MRJP1 expressing VSMCs was carried out. The cell numbers of MRJP1 expressing VSMCs at 24 h, 48 h, and 72 h were significantly reduced relative to controls (Fig. 2b). In addition, the time period required for cell numbers to double, or doubling time (*td*), was significantly delayed in MRJP1 expressing VSMCs (23.6 ± 3.2 h) compared to that of controls (15.9 ± 1.5 h) (Fig. 2c). Moreover, the expression level of proliferating cell nuclear antigen (PCNA) in MRJP1 expressing VSMCs was significantly reduced compared to controls by Western blotting analysis (Fig. 3b). This evidence suggests that the proliferative ability is inhibited by the expression of MRJP1in mouse VSMCs.

### Expressing MRJP1 in mouse VSMCs significantly alters proteome settings

To determine the proteome setting changes by the expression of MRJP1 in VSMCs, a state-of–the-art MS-based proteomics analysis was performed. In all, 6,380 proteins (3,058 protein groups) were identified in both control and MRJP1 expressing VSMCs shown in Table S1 (relevant peptides in Table S2), 675 of which were significantly altered in terms of their expression levels (fold change ≥1.5 and *p* < 0.05) due to the expression of MRJP1 in VSMCs. Notably, among those 675 differential proteins between the two groups, 646 were down-regulated in MRJP1 expressing VSMCs, whereas only 29 were up-regulated (Table S3). The expression levels of specific markers of VSMC and PCNA also show similar trends to the results from the Western blotting analysis; specifically, the expression levels of αSMA, calponin and PCNA were significantly down-regulated in MRJP1 expressing VSMCs in both proteomics and Western blotting analysis (Fig. 3a,b). SM22, or transgelin, was significantly down-regulated by Western blotting analysis (Fig. 3b) but was not included in proteomic data since it was missed in one sample. However, SM22 homolog, or transgelin-2, which has functions similar to SM22, was measured as a differential protein in proteomic analysis (Fig. 3a).

### Expressing MRJP1 in mouse VSMC induces down-regulation of pathways associated with blood pressure regulation via action upon VSMCs

**Figure 4:**
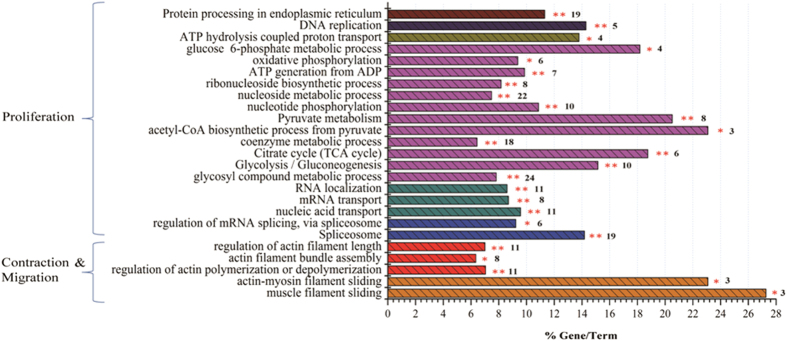
MRJP1 expression altered essential pathways related to VSMC contraction, migration and proliferation. Functional classes and biological pathway enrichment of differential proteins identified between control and MRJP1 expressing VSMCs. The signifcantly enriched pathways are analyzed by ClueGo, a Cytoscape plugin. *p* values < 0.05 were considered significant by a right-side hypergeometric algorithm and an FDR is done by Bonferroni step down test to correct the *p* value of the enriched terms. Columns shown the same colors are in the similar functional categories.

**Figure 5:**
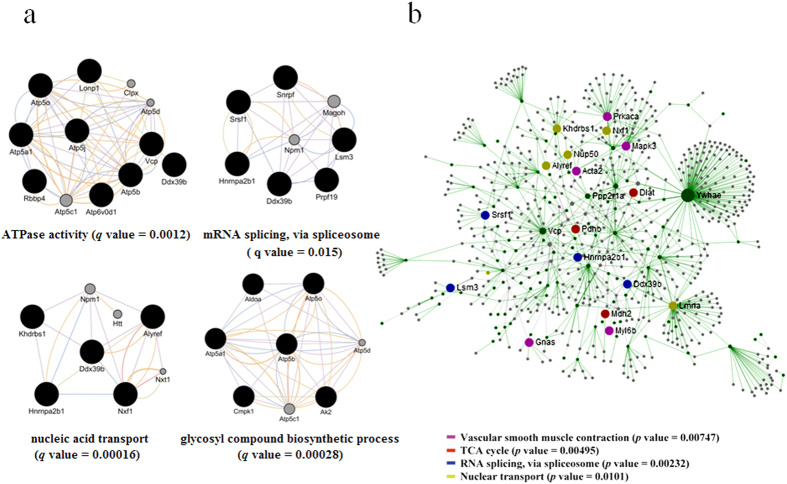
Protein-protein interaction (PPI) network of the down-regulated proteins in MRJP1 VSMC. (**a)** Represents the PPI networks of the down-regulated proteins are visualized by GeneMANIA plugin within Cytoscape. Networks are all enabled and the top 50 related genes and at most 20 attributes were displayed via GO biological process-based weighting. The terms are shown as “glycosyl compound biosynthetic process,” “ATPase activity,” “mRNA splicing, via spliceosome” and “nucleic acid transport,” respectively. *q* values < 0.05 are considered to be significantly enriched. **(b)** The node proteins that linked the network with high degree of interaction and the ones in pathways of “Vascular smooth muscle contraction,” “TCA cycle,” “RNA splicing, via spliceosome” and “Nuclear transport” were highlighted by NetworkAnalyst online software (http://www.networkanalyst.ca). Enrichment pathways are considered to be significant when *p* values < 0.05. The green nodes in the network are the down-regulated proteins by the expression of MRJP1.

**Figure 6:**
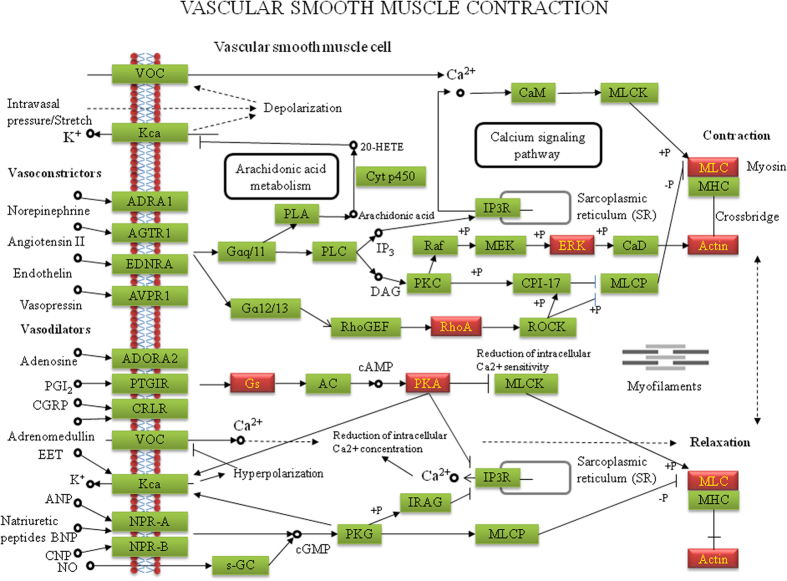
Differentially expressed proteins visualized in the pathway of Vascular smooth muscle contraction. Green labeled boxes represent the annotated proteins in the pathway of Vascular smooth muscle contraction. Highlighted red boxes indicate the differentially expressed proteins by the expression of MRJP1 in VSMCs that are enriched in the pathway. The proteins are visualized by online software KEGG Mapper (http://www.genome.jp/kegg/tool/map_pathway2.html) and the layout of the pathway is modified from that of Kanehisa Laboratories.

To understand the mechanisms of MRJP1 regulation to VSMC functionalities, bioinformatic tools were used to enrich differentially expressed proteins (Table S3) for specific functional groups and pathways. All 675 differentially regulated proteins (558 with GO annotation) were analysed. To distinguish whether the biological processes and pathways were functionally enhanced or suppressed, the up- and down-regulated proteins were separately analysed. Similar functional groups and pathways were enriched among those 675 differential proteins and 646 down-regulated proteins. The down-regulated proteins were enriched to pathways related to VSMC contraction, including muscle filament sliding (*p* = 0.028019) and actin-myosin filament sliding (*p* = 0.041833). The functional groups of actin filament activity relevant to VSMC contraction and migration were also significantly enriched. The leading term (the lowest *p* value) of actin filament activity was “regulation of actin polymerization or depolymerization (*p* = 0.000738).” Furthermore, pathways closely related to VSMC proliferation, including carbohydrate/nucleoside metabolism, RNA splicing, and RNA transport, were significantly enriched. The leading terms of these groups were “glycosyl compound metabolic process (*p* = 2.91 × 10^−10^),” “spliceosome (*p* = 1.51 × 10^−12^),” and “nucleic acid transport (*p* = 3.97 × 10^−5^)” respectively (Fig. 4 and Table S4). For the down-regulated proteins in the PPI networks, 109 terms were significantly involved (Table S5). Particularly, terms related to VSMC proliferation, including “glycosyl compound metabolic process (*q* = 0.00028),” “mRNA splicing, via spliceosome (*q* = 0.015),” “nucleic acid transport (*q* = 0.00016),” and ATPase activity (*q* = 0.0012) were significantly enriched (Fig. 5a). The information about such interactions that proteins could be connected out of the ones differentially expressed is shown in Table S6. In addition, the node proteins that linked the network with high degree of interaction and the ones in the pathways of “Vascular smooth muscle contraction,” “TCA cycle,” “RNA splicing, via spliceosome” and “Nuclear transport” were highlighted by NetworkAnalyst (Fig. 5b) and the pathways were visualized by KEGG Mapper (Fig. 6 and Figs S4–S6). The detailed pathway information is shown in Table S7. Additionally, the up-regulated proteins were significantly enriched in the pathway of “Mineral absorption” (*p* = 0.000387) (Table S4).

## Discussion

Hypertension is one of the major risk factors resulting in many CVDs and other diseases in humans^20^. RJ is a well-known natural product that has benefits for human health and has a purported function in resisting hypertension both by oral administration and by *in vitro* assay^16^,^17^. To investigate the specific functional components and working mechanisms of RJ proteins in regulating blood pressure, we created a model system by cloning the CDS of *mrjp1* into mouse VSMCs by the lentivirus, which can express MRJP1 in VSMCs. Using our established model, a wide range of functional analyses reveal that the expression of MRJP1 in mouse VSMCs plays key roles that may be associated with lowering hypertension via action on VSMCs. Further proteomics analysis delineates the potential blood pressure regulating mechanisms of introducing MRJP1 into VSMCs in mice. We found that a wide array of functional classes and pathways closely related to the contraction, migration, and proliferation in mouse VSMCs are functionally down-regulated by inhibiting muscle filament movement, energy, and genetical material supply, thus influencing blood pressure.

Many severe cardiovascular diseases, including hypertension and atherosclerosis, result from the abnormal contraction, migration and proliferation of VSMCs. In blood vessels, VSMCs maintain vascular tone and determine blood pressure via vessel constriction/dilation. The contractile VSMCs, which are non-proliferative and fully differentiated, are usually indicated by the expression of several specific marker genes at high levels, such as αSMA, SM22 and calponin^4^,^5^. In short, αSMA, the major and specific isoform of actin expressed in VSMCs, is responsible for VSMC contraction. An αSMA deficiency can result in decreased contractibility of VSMCs and hypotension in mice^21^,^22^. SM22 is a calponin-related protein that is specifically expressed in adult smooth muscle^23^, which is functionally important in maintaining the contractibility and mobility of VMSC^24^. Calponin, identified as thin filament-binding protein, is believed to serve as the contractile apparatus and cytoskeleton of SMC^25^. Hence, expression levels of such VSMC specific marker genes are key to determining the VSMC differentiating and contractile ability^26^. In our study, the down-regulated expression levels of αSMA, SM22, and calponin in MRJP1 expressing VSMC are indicative of the fact that MRJP1 has a potential to inhibit VSMC differentiation from proliferative type. Therefore, MRJP1 may be functionally important for reducing blood pressure by decreasing the VSMC contractile phenotype. Moreover, aberrant VSMC growth is also associated with hypertension and atherosclerosis. The higher proliferation and migration of VSMCs tend to constitute thicker blood vessel walls of higher lumen/media ratios, thus increasing the risk for such diseases^7^,^8^,^9^. Here, immortalized VSMCs growed faster (*td* = 15.9 ± 1.5 h) comparing to the rat primary VSMC (*td* = 21.4 ± 1.7 h)^27^, indicating they might be the better cellular model to simulate pathological conditions in blood vessels. The significantly down-regulated expression of PCNA and prolonged *td* by the expression of MRJP1 in VSMCs manifest that MRJP1 is able to reduce VSMC proliferation. In addition, the basal differentiating and proliferative abilities of VSMC inhibited by MRJP1 indicate that MRJP1 may not be a phenotypic switch between contractile and proliferative types of VSMC. It is reported that the over-expression or knockout/knockdown of certain genes or proteins in VSMCs can result in up or down-regulation of blood pressure^11^,^28^,^29^. However, most genes or proteins discussed above are endogenous molecules. Here MRJP1 is an exogenous protein transferred into VSMCs, suggesting that the encoding protein of MRJP1 in VSMCs has biological activities in mammalian cells. MRJP1 is the most abundant protein in RJ, which is secreted by the hypopharyngeal gland of nurse honeybee workers in order to feed larvae and queen bees^12^. As a highly nutritious and functional food, it imposes no toxic risks on VSMCs. Applying lentivirus as a vector is a widely-used method for generating genetically modified organisms. It is also applicable in gene therapy research. For example, salusin-β, a bioactive peptide involved in the pathogenesis of atherosclerosis, has been delivered into rats through intravenous injection of lentivirus, which has been successful in creating hypertension in rats^11^. Therefore, the introduction of MRJP1 into mouse VSMCs by the lentiviral vector makes MRJP1 inhibit the over-contracted, migrating or proliferative VSMCs, which could inspire new approach for remedying hypertension or atherosclerosis. Hence, the reported data here may bring a promising therapeutic potential to treat hypertension or atherosclerosis in humans using gene therapy from natural bee-products.

Blood regulatory function is largely dependent on VSMCs’ contractibility and migrating ability^30^, To understand the mechanisms of MRJP1 functionalities in regulating VSMC contraction and migration, proteome alteration between the control and MRJP1 expressing VSMCs was compared. The 646 down-regulated proteins were significantly enriched to a wide cascade of pathways associated with myosin-actin filament sliding, actin filament polymerization/depolymerization, and ATPase activity, suggesting they are essential in regulating VSMC contraction and migration. Generally, in the VSMC contractive process, calcium binds to calmodulin and then activates a myosin light chain kinase by phosphorylating myosin light chains. Then, a cross-bridge between myosin heads and actin filaments is formed^31^, and this sliding process results in VSMC contraction. During the phosphorylation process of myosin light chains, ATP is required to facilitate the phosphorylation to form a cross-bridge between myosin heads and actin filaments^32^. Here, the down-regulated proteins by expression of MRJP1 in VSMCs were significantly enriched to these pathways. For example, Myl6b is part of myosin light chains with the function of ATPase activity^32^,^33^ and αSMA is the actin filament. The reduced expression of these two proteins caused by expression of MRJP1 in VSMCs suggests that the cross-bridge is weakened, thereby diminishing the VSMC contraction. Moreover, actin monomers go through polymerization/depolymerization to cause elongation of actin filaments^34^,^35^, which engage in cell contraction and migration^36^,^37^. During this process, actin filament assembly is powered by ATP hydrolysis^34^,^35^. In our study, the down-regulated functions of pathways related to actin filament polymerization/depolymerization in MRJP1 expressing VSMCs indicate that actin filament formation is inhibited. Additionally, the actin filaments are assembled into bundles in the cells. This crosslink can strengthen the actin filaments and their tension, and increase ATPase activity of myosin as well^38^. Here, the significantly enriched pathway of actin filament bundle assembly by the down-regulated proteins in MRJP1 expressing VSMCs indicates that the VSMCs contractibility is decreased via reducing actin filaments, elongation, and bundle assembly. For example, ras homolog gene family, member A (RhoA) is engaged in the pathways of actin filament polymerization and actin filament bundle assembly, because it activates myosin phosphatase target subunit 1 and myosin light chain in VSMCs^39^. So, the down-regulated RhoA in MRJP1 expressing VSMCs suggests its importance in inhibiting VSMC contraction. ATPase catalyzes ATP hydrolysis to provide energy and phosphates to the donor molecules to promote phosphorylating processes within the cells^40^. The down-regulated proteins implicated in ATPase activity in MRJP1 expressing VSMCs imply that the lowered ATPase activity hinders the phosphorylation of myosin light chains, a crucial process prior to cross-bridge formation, thus inhibiting the VSMC contraction. Collectively, MRJP1 has the potential to reduce VSMC contractibility and migrating ability through the inhibition of muscle filament activities involving muscle filament sliding/myosin-actin filament sliding, actin filament polymerization/depolymerization and ATPase activity.

Energy metabolism is essential to cell proliferative ability^41^. This is reflected in our data— that the down-regulated proteins by MRJP1 expression were significantly enriched to energy metabolizing pathways in mouse VSMCs, such as glycolysis, citrate cycle and pyruvate metabolism, which are essential for ATP production. The declined energy supply within the cell definitely results in decreased VSMC growth^42^. For instance, dihydrolipoamide S-acetyltransferase (Dlat), an enzyme anchored in mitochondrion, catalyzes pyruvate to acetyl-CoA that is a basic necessity to the tricarboxylic acid cycle, the key process to produce ATP. The observed down-regulation of Dlat by the expression of MRJP1 signifies that the efficiency of ATP production is reduced and thus energy provision for cell growth is diminished. Given ATPase activity was reduced by MRJP1 expression, this can give rise to the decreased ATP utilization, and in turn hamper the growth of VSMCs. For the majority of cancer cells, fundamental energy metabolism changes to support high proliferation^43^. Recent reports show that VSMCs have increased rates of glycolysis in pulmonary artery hypertension^44^, indicating that there is close correlation between carbohydrate catabolism and hypertension. In addition, cell proliferation requires nucleosides to duplicate the genome. The fact that a large number of down-regulated proteins participated in the pathway of nucleoside metabolism suggests that nucleoside metabolism is minimized, thereby leading to the inhibitory effect of VSMC growth due to the decreased provision of nucleoside materials. For example, UMP/CMP kinase (Cmpk1) plays a pivotal role in the formation of UDP, CDP, and dCDP within cells, which are building blocks for cellular nucleic acid synthesis^45^. Therefore, down-regulation of Cmpk1 hinders genetic material supply for cell growth. It is well-known that cell maintenance and growth demand RNA biogenesis and transport. This is reflected in our data that the down-regulated proteins significantly enriched to the pathways of RNA splicing and mRNA nuclear transport. For example, serine/arginine-rich splicing factors play a range of essential roles in RNA splicing, including the prevention of exon skipping, ensuring splicing accuracy, as well as mRNA nuclear transport^46^,^47^,^48^. Flotillin-1 is reported to enhance cancer cell growth^49^, which was also enriched in the down-regulated pathway of RNA transport. Hence, the expression of MRJP1 in VSMCs reveals the significant roles in reduction of RNA splicing and transport, which inhibits VSMC growth. In all, it is clearly demonstrated that the expression of MRJP1 diminishes VSMC growth by down-regulating the metabolic processes of carbohydrate, nucleoside, and RNA splicing and transport, which ultimately result in the weakening of VSMC proliferation.

Proteins function cooperatively by interactions in the PPI networks in a living cell, and the investigation of these synergies is key to the understanding of biological activities in a systematic way. Here, the differentially regulated proteins by the expression of MRJP1 in VSMCs were linked to a PPI network and enriched to a number of pathways by the down-regulated proteins, such as ATPase activities and nucleic acid transport, which are related to VSMC proliferation. For instance, ATP Synthase, H+ Transporting, Mitochondrial F1 Complex, and Beta Polypeptide (ATP5b) are subunit of mitochondrial ATP synthase, which co-localize with valosin containing protein (Vcp), co-express, and share protein domains with ATP synthase, H+ transporting, mitochondrial F1 complex, alpha subunit 1(Atp5a1). They act and interact with each other to ensure the normal running of ATPase activities. Heterogeneous nuclear ribonucleoprotein A2/B1(Hnrnpa2b1) co-localizes with nuclear RNA export factor 1(Nxf1) and co-expresses KH domain containing, RNA binding, signal transduction associated 1(Khdrbs1). Such interactions are essential to the event of nucleic acid transport. These interactions by the expression of MRJP1 in VSMCs may reduce function of these pathways, thus suppressing VSMC proliferation. Hence, the PPI network analysis gives us further insight into the biochemical cascades that regulate blood pressure by the integration of MRJP1 into mouse VSMCs.

The mineral absorption pathway is functionally important for absorbing metal ions in cells. Metal ions are vital to the removal of free radical in cells^50^. Two up-regulated proteins by the expression of MRJP1, ferritin heavy chain polypeptide1 (Fth1) and metallothionein 1 (Mt1), were involved in the functionality of the mineral absorption pathway. It is reported that both Fth1 and Mt1 are functionally important for combining metal ions and removing free radicals, which decreases reactive oxygen species (ROS)^51^,^52^. ROS can modulate vascular cell growth, migration, and contraction^53^; therefore, the up-regulation of Fth1 and Mt1 may help reduce blood pressure by lowering ROS in VSMCs, and this in turn may help lessen blood pressure.

## Conclusion

In this study, we successfully cloned the CDS of *mrjp1* into the lentiviral vector followed by the production of the corresponding lentivirus. After the lentiviral transduction, mouse VSMCs are able to express proteins of MRJP1. In this mouse VSMC model that expresses MRJP1, the most abundant RJ protein, it is discovered that the contractile, migrating, and proliferative abilities of VSMCs are significantly reduced by the expression of MRJP1. This clearly demonstrates that MRJP1 may play a potential role in regulating blood pressure. To achieve the functionality in modulating blood pressure, the proteome setting has reshaped in VSMCs to fine-tune protein activity to optimize cellular behavior for lowering blood pressure. Thus the functionality of a wide repertoire of pathways involved in regulating VSMC contraction, migration, and proliferation, including myosin-actin filament sliding, actin filament polymerization, actin filament bundle assembly, ATPase activity, carbohydrate/nucleoside metabolism, and RNA splicing and transport are down-regulated. The reported data significantly expand the functional and mechanistic insights into blood pressure regulation by MRJP1. This provides sound clues and potential novel approaches for the treatment of hypertension and relevant cardiovascular diseases using genetically-engineered, natural products in gene therapy. In our next study, we would perform *in vivo* experiments that generate *mrjp1* transgenic mice followed by blood pressure measurement to test the functionality of MRJP1 in VSMCs of blood vessels. Our current cellular work is the first report that transferred the gene of the royal jelly protein, a well known bee-product with recognized health benefits, into the cell model to reveal potential avenues for regulating blood pressure by affecting VSMC functions. The reported data may be useful in treating hypertension with gene therapy using natural bee-products.

## Methods

### Chemical reagents

The chemical reagents were bought from Sigma-Aldrich (St. Louis, MO, USA). Otherwise the sources were specified.

### Mouse VSMC culture

Mouse (C57BL/6 strain) VSMC immortalized with SV40 large T antigen was purchased from American Type Culture Collection (CRL-2797, LOT: 60521753) and cultured in Dulbecco’s modified eagle medium (DMEM, GIBCO^®^, ThermoFisher Scientific, Waltham, MA, USA) containing 10% fetal bovine serum (FBS), under the condition of 37 °C and 5% CO_2_. Cells were dissociated by 0.25% trypsin-EDTA (GIBCO^®^) and thawed in DMEM containing 10% dimethyl sulfoxide (DMSO, Fisher Scientific, Pittsburgh, PA, USA) at −80 °C and further in liquid nitrogen. The thawed cells were revivified in a water bath at 37 °C precipitated at 1,000 r, 3 min to remove DMSO, then suspended and grown in the above-mentioned cell cultural media.

### Construction and genotyping of MRJP1 expressing VSMC

To construct the MRJP1 expressing VSMCs, the CDS of *mrjp1* was synthesized and cloned into the multiple cloning sites of the lentiviral vector (PLV-EGFP (2A) Puro), followed by lentivirus production according to the manufacture’s protocol (Inovogen Tech. Co., Beijing, PR China). Briefly, HEK293T cells were co-transfected by pLV-MRJP1 and helper plasmids for 48 h. The supernatant was centrifugated 25,000 g for 2 h at 4 °C to purify the lentivirus. The cultured VSMCs were then transduced with the lentivirus carrying *mrjp1* gene and screened by puromycin with the concentration of 2ug/ml. The EGFP fluorescence can be observed in the VSMCs after successful transduction by the lentivirus. In the mean time, VSMCs used for control were transduced with lentivirus that has no *mrjp1* gene expression but has puromycin resistance and EGFP expression. Subsequently, three independent EGFP controls and EGFP+MRJP1 clones screened by puromycin were used in the experiments. To genotype the MRJP1 expressing VSMCs, the CDS of *mrjp1* gene was amplified by PCR from the total cellular DNA and identified by agarose gel electrophoresis to ensure its integration in the VSMC genome (primers in Fig. S2a). Furthermore, the expression of *mrjp1* mRNA was detected and quantified by real-time RT-PCR, normalized by the mRNA of EGFP (EGFP, an indicator protein from the lentiviral vector, is visible in successfully transduced VSMCs; therefore, its mRNA level is suitable for the normalization of *mrjp1* mRNA expression), the real time RT-PCR procedure was described in “Real time RT-PCR analysis of mrjp1 mRNA expression.” Finally, the unique peptides of MRJP1 were detected in *mrjp1* expressing VSMCs by LC-MS/MS analysis to confirm the translation from *mrjp1* mRNA to MRJP1 protein. The LC-MS/MS analytical method was described in “Quantitative Proteomic Analysis of VSMC.”

### VSMC contractibility assay

Collagen gel was used to determine the VSMC contractibilities of both control and MRJP1 expressing VSMCs. The procedure generally followed the previously reported methods with minor modifications^5^,^54^. In brief, VSMCs were added to the collagen type I (Corning, NY, USA) from rat tail and cultured in a 24-well plate at 37 °C for 30 min (the mixture of 500 ul in each well containing collagen of 1.67 mg/ml and VSMCs of 1.2 × 10^5^). After the mixture (gel) was coagulated, the edge of the gel was separated by a pipette. Then DMEM containing 10% FBS was added to each well to culture for 72 h. The gel was photographed by a gel imaging system, and measured by NIH image J software (National Institute of Health, USA). The VSMC contractibility was determined by the contraction index (area of well – area of collagen gel/area of well), which was shown as mean ± S.D from three independent experiments with a *p* value < 0.05 considered significant by student’s *t* test. In addition, the expression levels of αSMA, SM22 and calponin were determined by Western blot for further verification of VSMC contractibility as described in “Western blotting.”

### VSMC migrating ability assay

The cell wound healing assay was performed to compare the migrating ability between control and MRJP1 expressing VSMCs. Briefly, 1 × 10^6^ VSMCs were planted in each well of the 6-well plate and cultured in DMEM containing 10% FBS at 37 °C and 5% CO_2_. Having been 100% confluent, the cells were scratched by a 200 ul pipette and immediately photographed. The wound distances were measured at 0 h, 6 h and 12 h. The migration indices were calculated as (D 0 h–D 6 h or 12 h)/D 0 h.

### VSMC proliferative ability assay

The cell number counting method was used to determine the VSMC proliferative ability between control and MRJP1 expressing VSMCs. Briefly, 1 × 10^5^ VSMCs were planted in each well of the 6-well plate and cultured in DMEM containing 10% FBS at 37 °C and 5% CO_2_. Cells were dissociated and then counted by using cell counting chamber after 24 h, 48 h and 72 h. Cell numbers were shown as mean ± S.D from three independent experiments with *p* value < 0.05 being considered significant by student’s t test. Additionally, *td* of VSMCs was determined using *ln(N*_*48h*_*/N*_*0h*_) = *k(t*_*48h*_ − *t*_*0h*_) and calculating *td* = *ln 2/k* when cell number doubling for a given k. The expression level of PCNA was determined by Western blotting as described in “Western blotting” for further verification of VSMC proliferative ability.

### VSMC immunofluorescence

Immunofluorescence was applied to further verify that αSMA was down-regulated in VSMCs with MRJP1expression. In brief, VSMCs, planted in a 24-well plate, grow to 70–80% confluent followed by fixation of 4% paraformaldehyde. The fixed cells were then treated with blocking buffer containing 3% bovine serum albumin and 0.1% Triton X-100. Afterwards, the cells were incubated with primary αSMA and secondary antibodies, and mounted with 4′,6-diamidino-2-phenylindole (DAPI) as well. The fluorescence was observed and photographed in an Olympus X71 microscope (Olympus, Tokyo, Japan). The fluorescent intensity was measured by NIH imageJ software (National Institute of Health, USA) to measure the expression levels of αSMA and DAPI. The αSMA/DAPI ratio was shown as mean ± S.D from three independent experiments with a *p* value < 0.05 considered significant by student’s *t* test.

### Real time RT-PCR analysis of *mrjp1* mRNA expression

To ensure that the CDS of *mrjp1* were able to be transcribed into the corresponding mRNA, real time RT-PCR analysis was applied to detect the *mrjp1* mRNA expression. To achieve this, pooled VSMCs growing in DMEM containing 10% FBS were collected for total RNA isolation using TRIZOL method. Briefly, VSMCs were homogenized in TRIZOL reagent followed by chloroform extraction and then centrifuged by 12,000 g for 15 min at 4 °C. The recovered supernatant was added to isopropanol to precipitate RNA by centrifuge at 12,000 g for 10 min. The isolated RNA was purified in 75% ethanol and dissolved in DEPC water to avoid RNA degradation. The total RNA was reversely transcribed to cDNA by using RevertAid First Strand cDNA Synthesis Kit (THERMO) following the manufacturer’s instructions. Then SYBR-Green (Applied Biosystems^®^) based real-time RT-PCR was conducted on a Bio-Rad CFX Connect™ system to quantify the mRNA level of MRJP1 (the primers in the Fig. S2b). The PCR protocol began at 95 °C for 3 min, followed by 40 cycles of 95 °C for 10 s, 55 °C for 20 s and 72 °C for 20 s; then the fluorescence was measured. Melting curve analysis was performed to ensure the specificity of the PCR product by heating from 65 °C to 95 °C in increments of 0.5 °C*/*s. The relative expression of mRNA in MRJP1 was normalized to that of EGFP. The mRNA expressions were quantified by −2^ΔΔCt^ method and were shown as mean ± S.D from three independent experiments with a *p* value < 0.05 considered significant by student’s *t* test.

### Western blotting

VSMCs at the logarithmic stage were lysated in a radioimmune precipitation assay buffer (Solarbio, Beijing, PR China) containing 1% phenylmethanesulfonyl fluoride as proteinase inhibitor. After precipitation in 13,000 g for 10 min, the supernatant containing proteins were collected and followed by denaturing in 100 °C with bromophenol blue. The protein samples were loaded to an 8% SDS-PAGE gel for electrophoresis to separate the proteins, and then transferred to the PVDF membranes. The membranes were blocked in 5% fat free milk to avoid non-specific bindings and incubated in primary antibodies against αSMA (BOSTER, Wuhan, PR China), SM22 (Abcam, Cambridge, UK), calponin (BOSTER, Wuhan, PR China), PCNA (Abcam, Cambridge, UK) and GAPDH (CWBIO, Beijing, PR China), followed by incubating in the corresponding secondary antibodies. The membranes were illuminated and photographed in the ChemiDoc imaging system (Bio-Rad). The bands were quantified by NIH imageJ software and the abundance level of proteins was normalized by GAPDH. The protein of interest*/*GAPDH ratio was shown as mean ± S.D from three separated experiments and *p* value < 0.05 was considered significant by student’s *t* test.

### Protein sample preparation for LC-MS analysis

The total proteins of both control and MRJP1 expressing VSMCs were extracted according to our previously described methods^55^. In short, VSMCs were ultrasonicated on ice and lysated in lysis buffer containing 8 M urea, 2 M thiourea, 4% 3-[(3- cholamidopropyl) dimethylammonio]-1-propanesulfonate (CHAPS), 20 mM Tris-base and 30 mM dithiothreitol (DTT), followed by centrifugation of 15,000 g for 15 min at 4 °C to obtain a protein supernatant. The proteins were precipitated by acetone for 30 min at −20 °C prior to being centrifuged twice at 15,000 g, 4 °C for 10 min. The precipitated pellets were redissolved in 40 mM NH_4_HCO_3_. The final protein concentration was quantified using a Bradford assay. The protein samples were reduced with a solution of 10 mM DTT for 1 h. Then the samples were incubated in 50 mM iodoacetamide for alkylation in the dark for 1 h. Proteins were digested using trypsin (sequencing grade, Promega) overnight at 37 °C in a 1:50 trypsin-to-protein mass ratio. The enzymatic digestion was stopped by adding 1 μL of formic acid. Finally, peptides were vacuum-dried using a SpeedVac system (RVC 2-18, Marin Christ, Osterod, Germany) for the following LC-MS analysis.

### Quantitative Proteomic Analysis of VSMC

To create the quantitative proteomic profiles of control and MRJP1 expressing VSMCs, peptide samples were re-dissolved in 0.1% formic acid and loaded onto an LC-MS system with three replicates. The EASY-nLC 1000 (Thermo Fisher Scientific, Bremen, Germany) nano liquid chromatography system was coupled with Q-Exactive plus (Thermo Fisher Scientific) via the nanoelectrospray source. Reverse-phase chromatography and trap column packed with 2 μm C18 (100 Å, 75 μm × 50 cm, Thermo Fisher Scientific) were used for peptide enrichment, and peptides were further separated on a column packed with 2 μm C18 (100 Å, 75 μm × 50 cm, Thermo Fisher Scientific) for analysis. The mobile phase buffer consisted of buffer A (0.1% formic acid in water) and buffer B (0.1% formic acid in acetonitrile). Peptides were separated at a flow rate of 350 nl/min using the following gradients: from 3 to 8% buffer B for 10 min, from 8 to 20% buffer B for 80 min, from 20 to 30% buffer B for 20 min, from 30 to 90% buffer B for 5 min, and 90% buffer B for 10 min. Ion signals were collected in a data-dependent mode with the following settings: full scan resolution at 70,000, scan range: m/z 300–1,800; MS/MS scan resolution at 17,500, isolation window: 2 m/z, normalized collision energy: 27, loop count 10, and dynamic exclusion was also used (charge exclusion: unassigned 1, >8; peptide match: preferred; exclude isotopes: on; dynamic exclusion: 10 s).

The MS/MS data were retrieved using Xcalibur software (version 2.2, Thermo Fisher Scientific). Subsequently, the extracted spectra were searched by in-house PEAKS software (version 7.0, Bioinformatics Solutions, Waterloo, Canada) against the mouse database generated from UNIPROT containing 76,079 protein sequences (released in August 2015). Precursor mass tolerance was set at 15.0 ppm, and fragmention tolerance was set at 0.05 Da. The following modifications were applied: carbamidomethylation (C)/+57.02 Da was selected fixed modification, and oxidation (M)/+15.99 Da was selected as variable modifications. The other parameters used were the following: enzyme, trypsin; allowing a nonspecific cleavage at neither end of the peptide; maximum missed cleavages per peptide, 2; maximum allowed variable PTM per peptide, 2. A fusion target-decoy approach was used for the estimation of FDR and controlled at ≤1.0% (−10 log P ≥ 20.0) at both protein and peptide levels.

Protein identifications were used only if at least two spectra were identified in one sample. The relative quantification was performed through the label-free approach in the Q module of PEAKS by using the expectation-maximization algorithm as described previously^56^. Peptide features and proteins (whose peptides detected in all 6 samples were selected) of fold change ≥1.5 and statistical *p* value < 0.05 were considered significantly different between groups. The LC-MS/MS data have been deposited to the ProteomeXchange Consortium (http://proteomecentral.proteomexchange.org) via the PRIDE partner repository with the data set identifier PXD004207.

### Bioinformatics analysis

To produce the differential protein profile between control and MRJP1 expressing VSMCs, the differentially expressed proteins (Table. S3) were clustered using uncentered Pearson correlation and average linkage by gene cluster 3.0 and visualized by Java Treeview software. Furthermore, to interpret the mechanisms of MRJP1 regulating VSMC functions, the down- or up- regulated protein lists with mouse Uniprot ID were analyzed by CluoGo v. 2.1.6, a Cytoscape plugin (http://www.ici.upmc.fr/cluego/)^57^ software through the functional ontology categories in biological process and KEGG pathways. The significances of the enriched pathways were calculated by a right-side hypergeometric algorithm and an FDR was done by Bonferroni step down test to correct the *p* value in the software, which is based on number of associated genes in this work and the total associated genes in the database of mice. The *p* value < 0.05 was significant. Additionally, to understand the biological significance of the identified proteins engaged in blood pressure regulatory activities in a systematic way, protein-protein interaction (PPI) networks were constructed by GeneMANIA^58^; the database includes genetic and protein interactions with the settings of all enabled networks and 50 displayed related genes using GO biological process based weighting. The networks were visualized by Cytoscape. Finally, the node proteins that linked the network with high degree of interaction and the ones in particular pathways were highlighted by NetworkAnalyst online software (http://www.networkanalyst.ca)^59^,^60^ and the online software KEGG Mapper (http://www.genome.jp/kegg/tool/map_pathway2.html) was used to visualize the pathways.

## Additional Information

**How to cite this article**: Fan, P. *et al*. Functional and Proteomic Investigations Reveal Major Royal Jelly Protein 1 Associated with Anti-hypertension Activity in Mouse Vascular Smooth Muscle Cells. *Sci. Rep.* **6**, 30230; doi: 10.1038/srep30230 (2016).

## Supplementary Material

Supplementary Information

Supplementary Dataset 1

Supplementary Dataset 2

Supplementary Dataset 3

Supplementary Dataset 4

Supplementary Dataset 5

Supplementary Dataset 6

Supplementary Dataset 7
