# Supplementary material for: Functional and Proteomic Investigations Reveal Major Royal Jelly Protein 1 Associated with Anti-hypertension Activity in Mouse Vascular Smooth Muscle Cells
Source: Sci Rep. 2016 Jul 22;6:30230. doi: 10.1038/srep30230 (PMC4957218; doi:10.1038/srep30230)
Supplement: Supplementary Information [file srep30230-s1.pdf]

Functional and Proteomic Investigations Reveal Major Royal Jelly Protein 1 Associated with Anti-hypertension Activity in Mouse Vascular Smooth Muscle Cells

Pei Fan[1,2,§], Bin Han[1,§], Mao Feng[1], Yu Fang[1], Lan Zhang[2], Han Hu[1], Yue Hao[1], Yuping Qi[1], Xiaozhen Zhang[2], Jianke Li[1,*]:

§These authors contributed equally to this work

* Corresponding author

1. Institute of Apicultural Research/Key Laboratory of Pollinating Insect Biology, Ministry of Agriculture, Chinese Academy of Agricultural Sciences, Beijing 100093, China

2. College of Biological Engineering, Henan University of Technology, Zhengzhou 450001, China

Supplemental Figures

Fig.S1 EGFP expression shows the successful lentiviral transduction for mouse VSMCs. (a). is the mode chart of lentiviral vector harboring *mrjp1* gene. **(b).** is the EGFP expression observed in both control and MRJP1 expressing VSMCs after lentiviral transduction.

Fig.S2 Primers for PCR amplification and real time RT-PCR analysis. (a). shows the forword and revered primers for PCR amplification of *mrjp1*. **(b).** displays the forword and revered primers for real time RT-PCR analysis of *mrjp1* RNA.

Fig.S3 Immunofluenscent analysis shows reduced αSMA expression by MRJP1 in VSMCs. Immunofluenscent analysis of α SMA expression between control and MRJP1 expressed VSMCs. The expression level is shown as α SMA /DAPI from three independent experiments (n=3, mean ±S.D.) and * indicates $p < 0.05$.

Fig.S4 Differentially expressed proteins visulized in the enriched pathway of RNA transport. Green labeled boxes represent the proteins annotated in the significantly enriched pathway of RNA transport. Highlighted pink boxes indicate the differentially expressed proteins mapped into the pathway.

Fig.S5 Differentially expressed proteins visulized in the enriched pathway of Splicesome. Proteins in green labeled boxes are the proteins invovled in the significantly enriched pathway of Splicesome. Proteins in highlighted pink boxes are the differentially expressed ones mapped into the pathway.

Fig.S6 Differentially expressed proteins visulized in the enriched pathway of Citrate cycle. Green labeled boxes represent the protein references annotated in the significantly enriched pathway of Citrate cycle. The highlighted pink boxes are the entries of differentially expressed proteins mapped into the pathway.

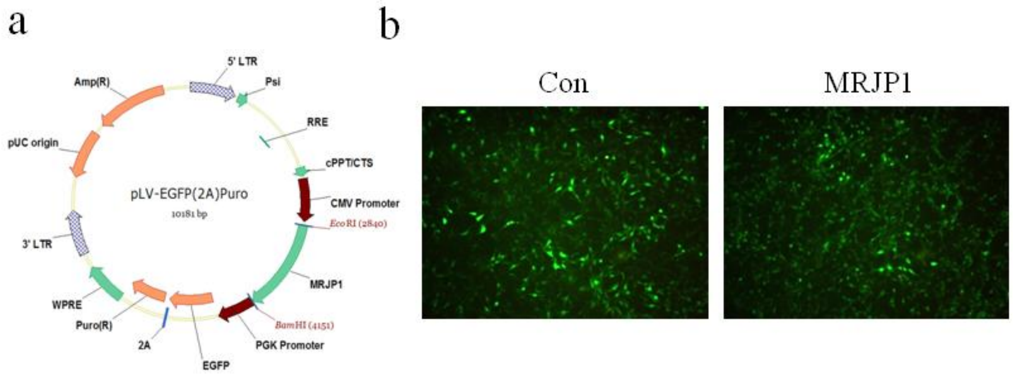

Fig.S1

a

Primers for PCR amplification

MRJP1-F: ATGACAAGATTGTTTATGCTGG

MRJP1-R: TTACAAATGGATTGAAATTTTG

b

Primers for Real time RT-PCR analysis

MRJP1-F: GTCCTGTAGCTTCCACCAGT

MRJP1-R: ATCCGAAGAAGAGAACGCCA

GFP F1: TATGGAGTGCAATGCTTCGC

GFP R1: TACTCCAGCTTGTGTCCCAG

Fig.S2

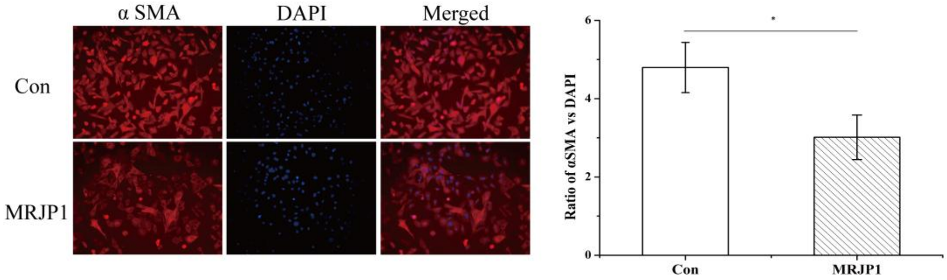

Fig.S3

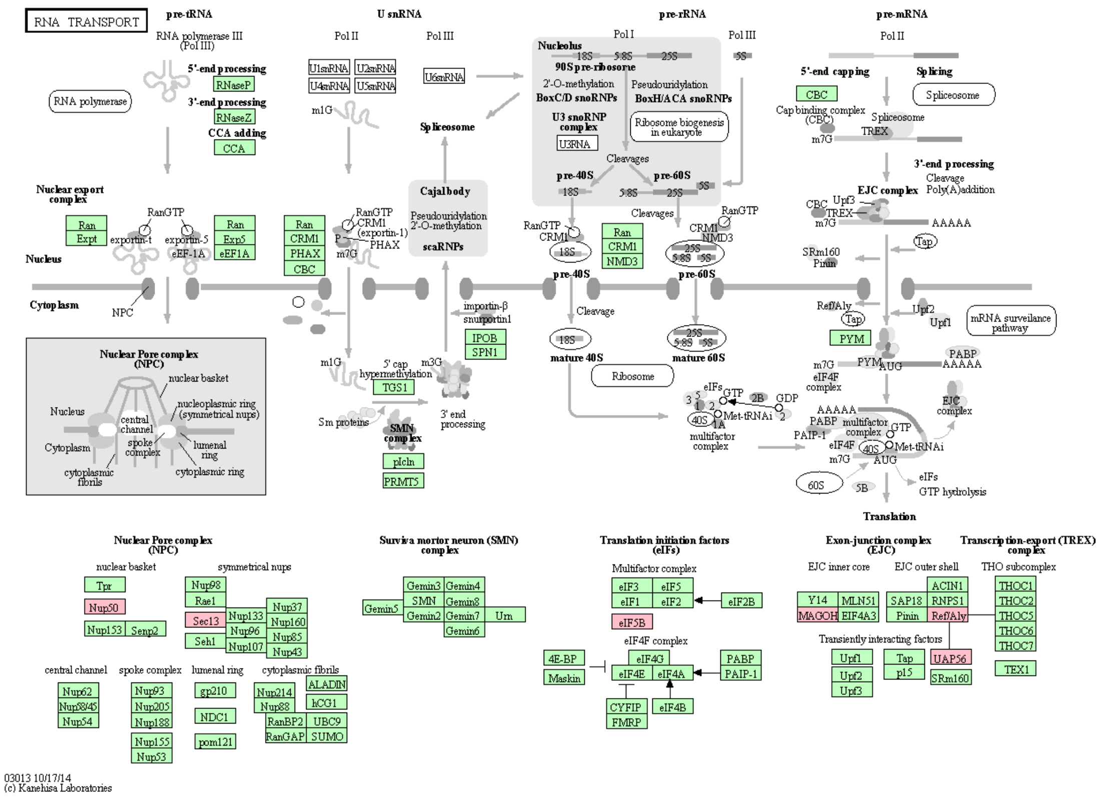
RNA TRANSPORT
pre-tRNA
RNA polymerase III (Pol III)
5'-end processing
RNaseP
3'-end processing
RNaseZ
CCA adding
CCA
RNA polymerase
U snRNA
Pol II
Pol III
U1snRNA
U2snRNA
U4snRNA
U5snRNA
U6snRNA
m1G
Spliceosome
pre-rRNA
Nucleolus
Pol I
18S
5.8S
25S
5S
90S pre-ribosome
2'-O-methylation
BoxC/D snoRNPs
Pseudouridylation
BoxH/ACA snoRNPs
U3 snoRNP complex
U3RNA
Ribosome biogenesis in eukaryote
Cleavages
pre-40S
pre-60S
pre-mRNA
Pol II
5'-end capping
CBC
Cap binding complex (CBC)
m7G
Splicing
Spliceosome
TREX
3'-end processing
Cleavage
Poly(A)addition
EJC complex
Upf3
AAAAA
Tap
SRm160
Pinin
Nuclear export complex
Ran
Expt
RanGTP
exportin-t
exportin-5
Exp5
eEF-1A
eEF1A
Nucleus
Cytoplasm
NPC
CRM1
PHAX
CBC
RanGTP
CRM1 (exportin-1)
Cajal body
Pseudouridylation
2'-O-methylation
scaRNPs
NMD3
importin-β
snurportin1
IPOB
SPN1
m1G
5' cap hypermethylation
m3G
TGS1
3' end processing
Sm proteins
SMN complex
pICln
PRMT5
Cleavage
mature 40S
mature 60S
Ribosome
Ref/Aly
Upf2
Upf1
mRNA surveillance pathway
PYM
AUG
PABP
eIF4F complex
eIFs
GTP
GDP
Met-tRNAi
multifactor complex
EJC complex
PAIP-1
60S
5B
GTP hydrolysis
Translation
Nuclear Pore complex (NPC)
nuclear basket
nucleoplasmic ring (symmetrical nups)
central channel
spoke complex
lumenal ring
cytoplasmic fibrils
cytoplasmic ring
Nuclear Pore complex (NPC)
nuclear basket
symmetrical nups
Tpr
Nup50
Nup153
Senp2
Nup98
Rae1
Sec13
Seh1
Nup133
Nup96
Nup107
Nup37
Nup160
Nup85
Nup43
central channel
spoke complex
lumenal ring
cytoplasmic fibrils
Nup62
Nup58/45
Nup54
Nup93
Nup205
Nup188
Nup155
Nup53
gp210
NDC1
pom121
Nup214
Nup88
RanBP2
RanGAP
ALADIN
hCG1
UBC9
SUMO
Survival motor neuron (SMN) complex
Gemin3
Gemin4
SMN
Gemin8
Gemin5
Gemin2
Gemin7
Unr
Gemin6
Translation initiation factors (eIFs)
Multifactor complex
eIF3
eIF5
eIF1
eIF2
eIF2B
eIF5B
eIF4F complex
4E-BP
Maskin
eIF4G
eIF4E
eIF4A
PABP
PAIP-1
CYFIP
FMRP
eIF4B
Exon-junction complex (EJC)
EJC inner core
EJC outer shell
Y14
MLN51
MAGOH
EIF4A3
SAP18
RNPS1
Pinin
Ref/Aly
ACIN1
Transiently interacting factors
Upf1
Upf2
Upf3
Tap
p15
UAP56
SRm160
Transcription-export (TREX) complex
THO subcomplex
THOC1
THOC2
THOC5
THOC6
THOC7
TEX1
03013 10/17/14
(c) Kanehisa Laboratories

Fig.S4

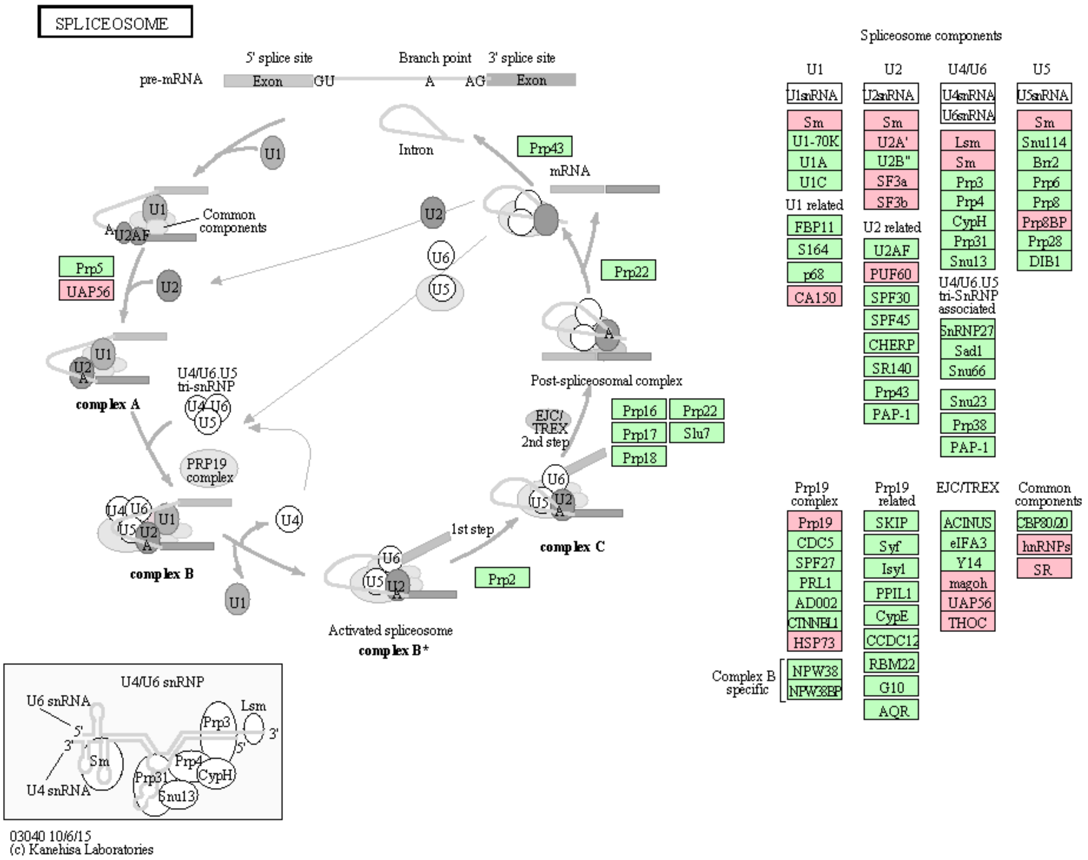
SPLICEOSOME
5' splice site
Branch point
3' splice site
pre-mRNA
Exon
GU
A
AG
Exon
U1
Intron
Prp43
mRNA
U1
Common components
U2AF
U2
U6
U5
Prp22
Prp5
UAP56
U2
U1
U2
complex A
U4/U6.U5 tri-snRNP
U4
U6
U5
Post-spliceosomal complex
Prp16
Prp22
Prp17
Slu7
Prp18
EJC/ TREX
2nd step
PRP19 complex
U4
U6
U1
U5
U2
complex B
U4
1st step
U6
U5
U2
complex C
Prp2
U1
Activated spliceosome
complex B*
U4/U6 snRNP
U6 snRNA
Lsm
Prp3
Sm
Prp4
Prp31
CypH
Snu13
U4 snRNA
5'
3'
03040 10/6/15
(c) Kanehisa Laboratories
Spliceosome components
U1
U2
U4/U6
U5
U1snRNA
U2snRNA
U4snRNA
U6snRNA
U5snRNA
Sm
U1-70K
U1A
U1C
Sm
U2A'
U2B"
SF3a
SF3b
Lsm
Sm
Prp3
Prp4
CypH
Prp31
Snu13
Sm
Snu114
Brr2
Prp6
Prp8
Prp8BP
Prp28
DIB1
U1 related
FBP11
S164
p68
CA150
U2 related
U2AF
PUF60
SPF30
SPF45
CHERP
SR140
Prp43
PAP-1
U4/U6.U5 tri-SnRNP associated
SnRNP27
Sad1
Snu66
Snu23
Prp38
PAP-1
Prp19 complex
Prp19
CDC5
SPF27
PRL1
AD002
CTNNBL1
HSP73
Prp19 related
SKIP
Syf
Isy1
PPIL1
CypE
CCDC12
RBM22
G10
AQR
EJC/TREX
ACINUS
eIFA3
Y14
magoh
UAP56
THOC
Common components
CBP80/20
hnRNPs
SR
Complex B specific
NPW38
NPW38BP

Fig.S5

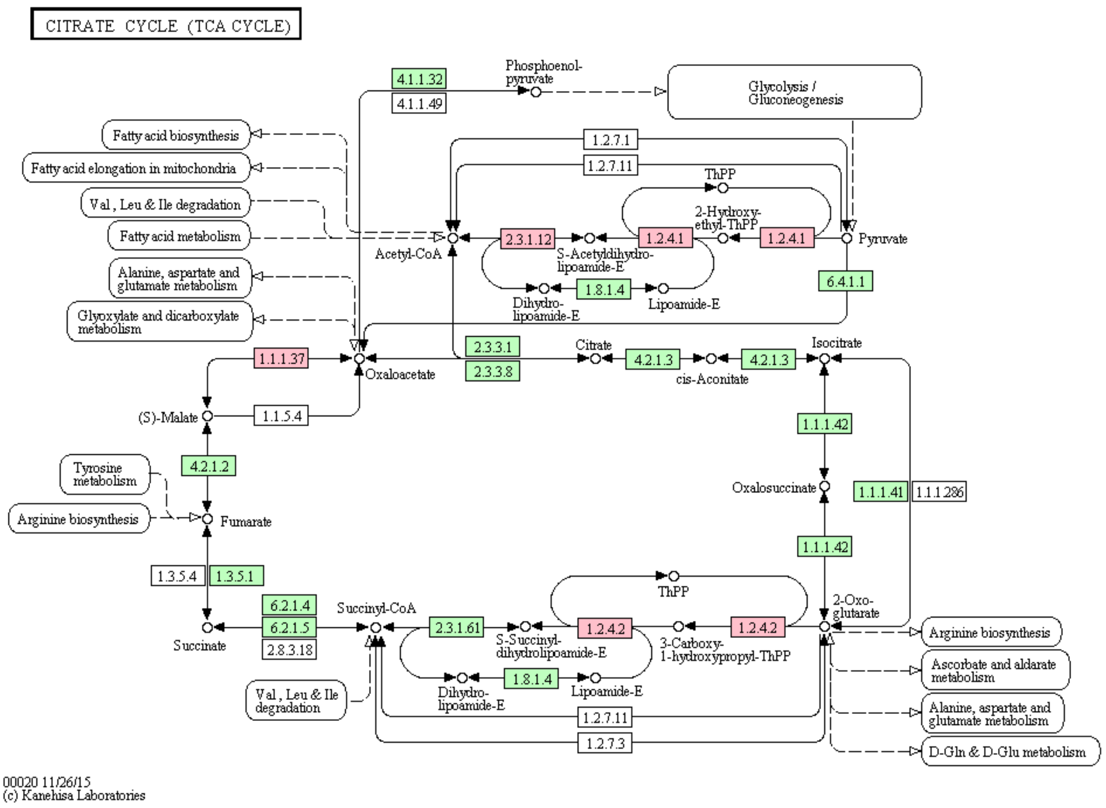
CITRATE CYCLE (TCA CYCLE)
4.1.1.32
4.1.1.49
Phosphoenol-pyruvate
Glycolysis / Gluconeogenesis
Fatty acid biosynthesis
Fatty acid elongation in mitochondria
Val, Leu & Ile degradation
Fatty acid metabolism
Alanine, aspartate and glutamate metabolism
Glyoxylate and dicarboxylate metabolism
1.2.7.1
1.2.7.11
ThPP
2-Hydroxy-ethyl-ThPP
2.3.1.12
1.2.4.1
1.2.4.1
Acetyl-CoA
S-Acetyldihydro-lipoamide-E
Pyruvate
1.8.1.4
Dihydro-lipoamide-E
Lipoamide-E
6.4.1.1
2.3.3.1
2.3.3.8
Citrate
4.2.1.3
4.2.1.3
Isocitrate
cis-Aconitate
1.1.1.37
Oxaloacetate
(S)-Malate
1.1.5.4
1.1.1.42
4.2.1.2
Tyrosine metabolism
Arginine biosynthesis
Fumarate
Oxalosuccinate
1.1.1.41
1.1.1.286
1.1.1.42
1.3.5.4
1.3.5.1
6.2.1.4
6.2.1.5
2.8.3.18
Succinate
Succinyl-CoA
2.3.1.61
S-Succinyl-dihydrolipoamide-E
1.2.4.2
1.2.4.2
3-Carboxy-1-hydroxypropyl-ThPP
2-Oxo-glutarate
Arginine biosynthesis
Ascorbate and aldarate metabolism
Alanine, aspartate and glutamate metabolism
D-Gln & D-Glu metabolism
Val, Leu & Ile degradation
Dihydro-lipoamide-E
1.8.1.4
Lipoamide-E
1.2.7.11
1.2.7.3
00020 11/26/15
(c) Kanehisa Laboratories

Fig.S6
